# Revisiting the argument from fetal potential

**DOI:** 10.1186/1747-5341-2-7

**Published:** 2007-05-17

**Authors:** Bertha Alvarez Manninen

**Affiliations:** 1 Arizona State University at the West Campus, Department of Integrative Studies, P.O. Box 37100, Mail Code 3051, Phoenix, AZ 85069, USA

## Abstract

One of the most famous, and most derided, arguments against the morality of abortion is the argument from potential, which maintains that the fetus' potential to become a person and enjoy the valuable life common to persons, entails that its destruction is *prima facie *morally impermissible. In this paper, I will revisit and offer a defense of the argument from potential.

First, I will criticize the classical arguments proffered against the importance of fetal potential, specifically the arguments put forth by philosophers Peter Singer and David Boonin, by carefully unpacking the claims made in these arguments and illustrating why they are flawed.

Secondly, I will maintain that fetal potential is morally relevant when it comes to the morality of abortion, but that it must be accorded a proper place in the argument. This proper place, however, cannot be found until we first answer a very important and complex question: we must first address the issue of personal identity, and when the fetus becomes the type of being who is relevantly identical to a future person. I will illustrate why the question of fetal potential can only be meaningfully addressed after we have first answered the question of personal identity and how it relates to the human fetus.

## Background

One of the most famous, and concurrently one of the most derided, arguments against the morality of abortion is the argument from fetal potential. This argument maintains that the fetus' potential to become a human person and enjoy the valuable life common to human persons entails that its destruction is *prima facie *morally impermissible. It is important to note here that the term "person" is used here in the strict philosophical sense; it is not meant to denote any and all human beings, as it is normatively used, but rather any being, human or nonhuman, that has the mental capacity to be rational, self-conscious, autonomous, and a moral agent.

One of the reasons that this argument is so interesting is that it is simultaneously ridiculed by some philosophers, and lauded by others. Many who reject the argument do so because they believe that it results in what is often called the "sperm/ova problem": if we regard the potential of a fetus to become a person as a morally relevant reason against killing it, we must also hold the same of human gametes, who also possess the potential to become persons. For example, Bonnie Steinbock writes:

The strongest objection to the argument from potential is that it seems to make contraception, and even abstinence, prima facie morally wrong. If the objection to abortion is that it deprives the zygote of a "future like ours," why, it maybe asked, cannot the same complaint be made of contraceptive techniques that ill sperm, or prevent fertilization? Why don't gametes have "a future like ours"?... so if abortion is seriously wrong because it kills a potential person, then the use of conceptive is equally seriously wrong. In using spermicide, one commits mass murder! [1]

L.W. Sumner makes a similar accusation:

... as far as the potentiality argument is concerned, abortion and contraception are both wrong, both equally wrong, and both wrong for precisely the same reason [2].

Conversely, other philosophers hold that the argument from potential is significant because it is the only thing that explains the stewardship that adult human beings have in regard to human neonates. Newborn infants lack the psychological maturity to possess goals, aims, beliefs, or purposes. This does not, however, exclude them from the moral community. The reason why it does not is because we realize that infants have the potential to develop these conscious goods, and it is this potential that, as Jim Stone argues, grounds the infant's interest in growing up and realizing that potential [3]. Every single semester that I teach the issue of abortion in class, I put up a picture of two cells that look striking similar, almost identical. I then reveal to my students that one is a skin cell, and the other is a fertilized egg at the zygotic stage of development. "Do they have the same moral status"?, I ask them. When I scratch my arm and kill skin cells, is my action as morally problematic as abortion? My students always answer that the two cell types are morally different; that the zygote is of a different status than my skin cells. In defense of this distinction, they always give the same reason: the zygote, if implanted into a uterus, has the potential to become a baby who will then become a person, whereas my skin cells do not. Since the vast majority of my students, in my seven years of teaching, share this intuition, I think that it is an intuition that is worthy of being explored rather than cavalierly dismissed.

The term "potential" as it is being used in this essay is not meant to describe mere *possibility*, i.e., X has the potential to achieve Y does not just mean X may possibly attain Y. If that were what was meant by potential, it would be very weak indeed. A seed would not just be a potential flower or plant, but also a potential food or a potential material for an art project. A kitten would not just be a potential cat, but also a potential delicacy at some restaurant, or a potential fur coat. Rather, potential, in the way I am using it here and the way I assume most advocates of the argument from potential use the concept, refers to, as Stephen Buckle puts it, a certain being's "potency... the power it [actually] possesses in virtue of its specific constitution" [4] to grow into a being of a certain sort. That is, X is a potential Y if X possesses the power to become Y; that X *will *become Y, if it lives long enough. In this way, a caterpillar is a potential butterfly (since it possesses the power to become a butterfly; it *will *become a butterfly if it lives long enough), as a child is a potential adult. A fetus is a potential person in this way; a fetus may not just *possibly *become a person, it *will *become a person, if its growth is unfettered and if it lives long enough.

With this introduction in mind, I can proceed to stating the goal of this paper, which is two-fold. First, I will criticize the classical arguments proffered against the importance of fetal potential, specifically the arguments put forth by philosophers Peter Singer and David Boonin, by carefully unpacking the claims made in these arguments and illustrating why they are flawed. I will argue that both Singer and Boonin assume that possessing actual personhood is a necessary condition in order to be accorded the right to life, but it seems that this is the very issue at hand and the very claim that those who argue in favor of the importance of fetal potential will challenge and subsequently reject.

Secondly, I will maintain that a fetus' potential can be salient when it comes to the morality of abortion, but that its proper place cannot be found until we first address a very important and difficult issue: the question concerning personal identity, and when the fetus becomes the type of being who is relevantly identical to a future person. I will illustrate how we cannot begin to tackle this perennial applied ethics issue without first addressing the metaphysical quagmire of personal identity, for where one stands on this metaphysical issue may greatly influence when one thinks potential begins to matter when it comes to the human fetus.

## Against Singer and Boonin

### What is wrong with the classical objections concerning fetal potential?

John T. Noonan uses a version of the argument from potential in order to provide one of many reasons why he deems abortion morally impermissible.

If a fetus is destroyed, one destroys a being already possessed of the genetic code, organs, and sensitivity to pain, and one which had an eighty percent chance of developing further into a baby outside the womb who, in time, would reason... once conceived, the being was recognized as man because he had man's potential [5].

For many philosophers, the argument from potential is considered invalid, either because the argument rests on a logical mistake or because it is misapplied in the abortion debate. Individuals who argue in the first manner maintain that the logical mistake comes in asking us to treat a potential X as if it is an actual X. Particularly, it asks us to treat a potential person, the fetus, as if it were already an actual person. However, the argument goes, how we treat a being ought to depend on its *actually possessed *properties, rather than on its *potentially possessed *ones. For those who would counter that an exception should be made for the life of the fetus because the right to life is somehow different and deserves to be extended to the fetus in virtue of its potential to become a person, the second objection applies. That is, given that potential Xs are never treated as actual Xs simply in virtue of their potential, there seems to be absolutely no reason to extend this special treatment to fetuses. As Paul Bassen writes: " [n]owhere outside the abortion debate itself is there a precedent for supposing that future prospects can create a present sake [6]." Therefore, although a standard human fetus possesses the potential to become a person and hence enjoy the kind of life typical to persons, this alone, the argument goes, does not create an obligation to protect the fetus as it exists currently.

It seems to me that those who argue against the moral relevance of fetal potential (whom I will call "anti-potentialists") do not really comprehend the role that their opponents give to the potential of the fetus. The fault of this misapprehension lies on both sides, for those who argue that the potential of the fetus does play a significant role when determining the morality of abortion (whom I will call "pro-potentialists") have not adequately clarified their position in light of the objections offered by anti-potentialists. It is my objective in this section to detail the anti-potentialists' concerns and propose some methods that the pro-potentialist can use in order to respond to these concerns.

Let us begin by outlining the standard argument against the moral permissibility of abortion given the purported moral relevancy of the fetus' potential.

**Premise 1**: All innocent persons have a moral right to life.

**Premise 2**: Since all innocent persons have a moral right to life, all potential innocent persons also have a moral right to life.

**Premise 3**: The human fetus is a potential innocent person.

**Conclusion**: The human fetus has a moral right to life.

For both anti- and pro-potentialists, premise one is uncontroversial, and premise three ought not to pose much of a problem either, since it is a biological claim rather than a moral one. Indeed, the standard human fetus, barring any unfortunate accident, will grow into an infant, child, and adult, and therefore will enjoy the life of a standard human person. The controversy, then, seems to lie in premise two: why is it true that the right to life of an innocent person ought to extend also to the fetus given its potential? How should premise two be defended?

Peter Singer presents a scathing objection to premise two because he does not think that an adequate defense of it is possible.

There is no rule that says that a potential X has the same value as an [actual] X, or has all the rights of an X. There are many examples that show just the contrary. Pulling out a sprouting acorn is not the same as cutting down a venerable oak. To drop a live chicken into a pot of boiling water would be much worse than doing the same to an egg. Prince Charles is the potential King of England, but he does not now have the rights of a king [7].

David Boonin offers the following analysis, and subsequent rejection of, premise two.

Perhaps the simplest argument from potentiality is one that rests on a general assumption of the following sort. Potential possession of a right entails actual possession of a right... [the argument's] major assumption rests on a logical error. It is certainly not true of properties in general that if a given individual potentially has a given property, then the individual already has this property [8].

As anti-potentialists regard it, any argument from potential commits the error of maintaining that for all things X, if X is a potential Y, then X ought to already be treated as an actual Y. Particularly, when it comes to the role of potential in the abortion debate, the argument commences with the uncontroversial premise that all innocent persons have a right to life. From this, the argument implicitly accepts the generalization expressed above. Since all actual innocent persons have a right to life (as premise one asserts), and since any potential person ought to be treated as an actual person (as entailed by the above generalization), a potential person also ought to possess the right to life. The fetus can certainly be substituted for the X variable, since a fetus is a potential person. Thus, the fetus also has a right to life.

At first blush, it does seem that Singer and Boonin present valid criticisms of this argument: it does not seem to follow that potential Xs are to be treated like actual Xs merely in virtue of their potential. Indeed, there are various examples that illustrate just the opposite. Prince Charles and Prince William are both potential Kings of England. Notice that they are not just *possibly *the future Kings of England (like perhaps Prince Harry is); if they live long enough, both Charles and William *will*, respectively, become the King of England in the future. However, this potential does not accord them the rights of kings while they are princes. Medical students, although potential physicians, do not possess the same rights (or responsibilities) as actual physicians do [9]. Children, although potential adults, do not possess the same rights as actual adults, for example, they cannot vote or drink alcoholic beverages. As these examples illustrate, beings are treated in accordance with their actually possessed properties, not their potentially possessed ones.

In light of this, the pro-potentialist seems to be making a mistake in logical reasoning, or at the very least asking us to make an exception when it comes to the human fetus without justification. But, as Singer puts it: "We should not accept that a potential person should have the rights of a person, unless we can be given some specific reason why this should hold in this particular case [10]." Singer argues that, when it comes to the right to life, it is essential that an individual be able perceive of herself as a continuing entity existing over time so that she may be capable of desiring continued existence. In order to possess this conceptual capacity, the being in question must be a person.

[T]he desire relevant to possessing a right to life is the desire to continue existing as a distinct entity. But only a being who is capable of conceiving herself as a distinct entity existing over time – that is, only a person – could have this desire. Therefore only a person could have a right to life... an individual cannot at a given time – say, now – have a right to continued existence unless the individual is of a kind such that it can now be in its interest that it continue to exist... since no fetus is a person, no fetus has the same claim to life as a person... [11]

Since the fetus never meets this necessary requirement, no fetus can ever be classified as a person, and its mere potential to one day be one does not ground a right to life now: "if [the requirements for personhood] are the grounds for not killing persons, the mere potential for becoming a person does not count against killing [12]."

It is in Singer's response that we can begin to see a possible line of defense for the pro-potentialist. Notice premise two again.

**Premise 2**: Since all innocent persons have a moral right to life, all potential innocent persons also have a moral right to life.

Now, also take notice of the different examples that are given by both Singer and Boonin in order to challenge the truth of premise two: Charles and William, although potential kings, do not have the same rights as an actual king. Medical students, although potential physicians, do not have the same rights as actual physicians. Children, although potential adults, do not have all the same rights as actual adults, for example they lack the right to vote or drink alcoholic beverages (this is my own example, although Boonin makes the broad argument that there are some rights and responsibilities that adults have that children do not). Thus, in order to be consistent with these examples, fetuses, although potential persons, are not persons yet and so ought not to be accorded a right to life.

What do all these examples have in common? What these examples all share is that the rights that are mentioned (the rights of the King of England, the rights of a physician, and the right to drink and vote) are such that it is sufficient that one meet an actual condition (of being a king, a physician, or an adult) in order to attain the corresponding right *and also that it is necessary that one meet this condition*. That is, it is necessary that one actually *be *the King of England in order to have the rights of the King of England, which is why neither Charles nor William possesses these rights currently. It is necessary that one *be *a physician in order to have the rights of a physician (which is why potential physicians do not have these rights, no matter how close to graduation they are). In our society, it is necessary that one be an actual adult in order to have the right to vote and drink alcoholic beverages. *This *is the reason why potential does not count it in these examples. If it is *necessary *to actually have a certain property in order to have a certain right, then it follows, of course, that potentially having this property cannot bestow that right upon an individual, for the individual simply does not meet the necessary requirement. Singer assumes that being a person is sufficient *and necessary *in order to have a moral right to life, and so he concludes that the pro-potentialist is mistaken if she thinks that having the potential to be a person can secure such a right. And Singer is correct, *if *it were also the case that being an actual person is a necessary condition for being accorded a moral right to life.

But I suspect that this is *exactly *what the pro-potentialist would (and should) contest. The pro-potentialist can agree that there are indeed cases where potential Xs are not to be treated as actual Xs solely in virtue of their potential, but she can also argue that this rule is not categorical. When it comes to the right to life, the pro-potentialist ought to question a much more fundamental assumption that is implicit in the anti-potentialist's argument: *Why *should being an actual person be a necessary condition for possessing a right to life? Singer's reasoning for this is unsatisfactory. He argues that self-consciousness, a mental capacity attributable only to persons, is necessary in order to be able to conceive of oneself as a distinct entity that exists over time, which in turn allows one to have the capacity for the requisite desire in order to possess a moral right to life: the desire for continued existence. In other words, Singer argues, *pace *Michael Tooley, that one must be capable of desiring continued existence in order to have an interest in, and thus a moral right to, continued existence [13]. I will illustrate the flaws of this argument below, but suffice it to say, for now, that the pro-potentialist should be wary of accepting this argument for why being a person is a necessary condition for possessing a moral right to life.

Perhaps, the pro-potentialist may argue, there are certain reasons why we bestow the right to life upon an actual person that may also apply to a potential person (and indeed there are, as I will argue below), thereby undermining the anti-potentialist's implicit assumption that it is necessary that one be an actual person in order to have a right to life. In other words, the pro-potentialist can agree that it is indeed true that potential Xs cannot have the same rights as actual Xs *providing that it is also the case that it is necessary that one be an actual X in order to have those rights*. However, the pro-potentialist can simply reject the thesis that it is necessary that one be a person in order to have a right to life (although being a person is certainly sufficient for such a right) and argue, instead, that the fetus' potential somehow suffices to extend to it a right to life [14]. Singer's and Boonin's examples will not convince the pro-potentialist that a fetus lacks a moral right to life because they are assuming a crucial premise that the pro-potentialist already rejects, if only tacitly.

### When potential is morally relevant

The task that the pro-potentialist now faces is to explain why the fetus' potential is a morally relevant characteristic that justifies extending to it a right to life. I believe that this can be done. What I want to do now is refute Paul Bassen's point that " [n]owhere outside the abortion debate itself is there a precedent for supposing that future prospects can create a present sake [15]" by discussing examples that seems to run contrary to this assertion.

#### The moral right to health insurance

Despite the rising costs and ailing benefits in our health care system, many would agree that all human beings, no matter how young, how old, or how sick, have a moral right to health insurance and the guarantee of health care. The Universal Declaration of Human Rights upholds this right in Article 25:

Everyone has the right to a standard of living adequate for the health and well-being of himself and of his family, including food, clothing, housing and medical care and necessary social services... [16]

In our society, then, most would maintain that currently ill individuals have a moral right to health insurance because having such insurance constitutes a great benefit for them and depriving an ill individual of medical insurance constitutes a harm. To deprive actually ill individuals of health insurance would make it increasingly difficult for them to get the treatment they need to alleviate their illness. In a society such as ours that seeks to protect the well-being of its citizens, the right to health insurance and subsequent medical care is certainly vital in order to achieve such protection.

In addition, *potentially *ill individuals, like my child or myself, also have a moral right to health insurance. But why do we accord this latter group health insurance, even though they are not *actually *sick? Because possessing health insurance, even in the absence of an impending illness, also constitutes a great benefit for potentially sick people and a deprivation of health insurance also constitutes a harm for potentially sick people.

Both actually sick people and potentially sick people possess what Joel Feinberg would call a "welfare interest" in continued health. Welfare interests are very basic or foundational interests; interests which, if thwarted, can result in an entire collapse of one's whole matrix of interests. Without continued health, the ability one has to fulfill other interests in life will be compromised. While I have an interest in continuing to work, raising my children, and taking care of my home, I would fail to realize any of these interests if my interested in health were thwarted. As Feinberg notes: " [a]ll the money in the world won't help you if you have a fatal disease... [17]." Welfare interests, which includes the interest in continued health, are "the very most important interests a person has, and cry out for protection, for without their fulfillment, a person is lost... an invasion of a welfare interest is the most serious... harm a person can sustain [18]."

Although a discussion concerning the exact purpose and nature of moral rights is beyond the scope of this paper, it suffices to say that one function of moral (and legal) rights is to protect people's interests, especially the very important and foundational ones. Actually sick individuals have a moral right to health insurance because it reasonably guarantees health care, and this, in turn, helps to protect their welfare interest in continued health. This is also what having health insurance provides for potentially sick individuals. Possessing health insurance gives potentially sick people peace of mind and a guarantee that when they do actually get sick medical treatment will be available for them. For consider what would occur if potentially ill people had to wait until they were actually sick to start the process of obtaining health insurance. By the time all the paperwork is completed and the monetary costs of premiums have exchanged hands, the illness will have probably taken a turn for the worst, and this, of course, would impede their respective welfare interest in continued health. So the very fact that people are potentially sick is a sufficient reason to extend health coverage to them now, even though there is no actual illness impending. Of course, a potentially ill person does not have the moral right to actual medical *treatment *while she is just potentially sick; she has this particular moral right only when she becomes actually sick. This is because being actually sick is a necessary condition to receiving actual medical care, since medical care does not serve any beneficial role to someone who is only potentially sick and a deprivation of medical care would not harm someone who is only potentially sick, only to those that are actually sick (e.g., there is no point in a doctor wrapping my arm in a cast if it is not broken in the first place). What a potentially sick person has a moral right to, even in her healthy state, is the guarantee that when she does become ill, health care will follow, and this is precisely what health insurance provides for the potentially sick person.

The welfare interest in continued health seems to be a wholly objective interest, rather than a subjectively mediated one. By this I mean that it is not necessary that a person actually desire continued health, or even possess the capacity to desire continued health, in order to possess an interest in it. An infant with a heart defect, for example, surely has an interest in a life-saving operation to repair her ailment, even though the infant lacks the cognitive capacities necessary to be capable of desiring the operation. While an infant cannot *take *an interest in her continued health (i.e., she is incapable of desiring her continued good health, since she lacks the capacity to even understand what "health" means), she surely *has *an interest in her continued health. It is because we recognize the importance of preserving the well-being of members in society, even if they are too rudimentary in their capacities to actually take an active interest in their health, that we extend a moral right to health care to the actually sick and the potentially sick. Whether one possesses the actual property of being sick, or whether one merely potentially possess that property, having a moral right to health insurance helps to secure one's important welfare interest in continued health, which, in turn, helps the development and well-being of members of our moral community in a variety of ways.

#### The moral right to an education

As a professor, I am exposed every day to the effects of sub-par education, especially when it comes time for students to turn in papers and their poor writing abilities become painfully apparent. This illustrates that being deprived of a good education works against the interest of a person. Thus, all *actually *rational agents have the moral right to a decent education because such an education hones their rational abilities and enriches their mind. To deprive a rational agent of an education constitutes a harm for her, since it deprives her of the opportunity to cultivate her rational faculties. To provide a rational agent a decent education, of course, constitutes a great benefit.

A very young, not fully rational, child, also has a moral right to a decent education even though she is not currently the rational agent she will become in the future. In fact, young children rarely practice many of the subjects that are taught to them at their young age, rather they come to exercise such knowledge sometime later in life. As R.J. Gerber puts it, "the careful education tendered to the young in our society suggests that we do, in fact, prize human potential for what it may actually receive in the distant future [19]." For example, there is very little a child of ten can do with her knowledge concerning long division or multiple digit multiplication (indeed, they are keen enough to pick this up to a certain extent when they cry: "When will I ever use this in real life?"). If pressed, neither parents nor teachers may think that the child is deriving such benefits from these facets of education currently. Rather, it is the child's *potential *as a future rational being that grounds her *current *interests in learning more complex mathematics. Therefore, it is indeed a good thing to allocate to her, indeed to all children, a moral right to a decent education, even though the fruits of such an education may not make themselves manifest until much later; to not do so runs the risk of impeding the child's capacities as a rational agent and thus stifles her well-being.

The interest in a good education may not be as foundational as the interest in continued health (for many people do live their lives and pursue other interests even in absence of a good education), but it is a very important interest nevertheless, and it is an interest that, also, is wholly objective rather than subjectively meditated. That is, even if a young child begs and screams not to go to school (as I often did as a child), we do not thereby conclude that she has forfeited her moral right to an education. Furthermore, an autistic child, or a child afflicted with Down syndrome, may not have an actual desire for an education and may even, because of limited cognitive capacities, wholly lack the capacity to possess such a desire. Yet if that education helps her advance her limited abilities so that she may become reasonably self-sufficient in the future, then she does *have *an interest in the type of education that would benefit her even if she cannot *take *an interest in it. This all illustrates, again, that the interest in obtaining an education really is an objective, rather than a subjective, interest.

Consider the current market for educational infant toys. Many parents shower their children with educational toys from infancy (the Baby Einstein and Leap Frog collections attest to the fact that there is a viable market in this area), given the parents' desire to cultivate and nurture their child's innate rational abilities from early life. Even the best possible pet owners, in contrast, do not expose their cats or dogs to such educational toys simply because these nonhuman animals lack the potential to achieve a rational mind and so the animals would derive absolutely no benefit from this exposure in any way. This is why, I suspect, there are no Baby Einstein or Leap Frog collections for puppies or kittens. This certainly attests to the fact that we treat beings with certain potentials (e.g. the potential for rational agency or autonomy) differently than we treat beings that lack such potential. As L.W. Sumner notes:

It is not astonishing that someone who values rationality should care for creatures who will be rational in the future as well as those who are rational at present. Protecting the lives of the potentially and actually rational are merely two different means of promoting rationality [20].

#### Other instances when potential matters

To depart from the subject of rights briefly, consider moral education. We teach our very young children to say "please" and "thank you," to share their toys, to say that they are sorry when they have done something wrong. When we ask this of a two-year-old now, we do not do so because she is receiving current benefits from such moral education. A two-year-old will probably not learn an immediate lesson about the importance of sharing or politeness when we make her share her toys or express gratitude, rather we do so because of her potential as a future moral agent, and her potential in this area certainly affects how we treat her *now*. Indeed, if one sides with Aristotle on this matter, it is imperative that we bestow moral education upon our children from a very early age, even though they may not be current moral agents, because this will make a crucial difference towards their becoming virtuous individuals in the future: "It is not unimportant, then, to acquire one sort of habit or another, right from our youth; rather, it is very important; indeed, all-important [21]." That is, a child's potential to become a moral agent grounds a present interest in being exposed to moral education.

Finally, consider a young child that displays a keen ability to play a musical instrument. Her music teacher tells you, her parent, that she has the potential, if that potential is cultivated, to become a fantastic pianist or violinist. Her potential to be a great musician creates a current interest in being given the best music lessons that are within your financial means to acquire. If the child is deprived of such lessons, and as a result never cultivates or realizes her potential, then it can be said, quite rightly, that the child has been harmed, and that she would have greatly benefited had she received those lessons. The child's *potential *grounds a *current *interest in music lessons, and her potential also grounds the extent that she is harmed if she is deprived of those lessons (i.e., if her potential to become a phenomenal musician was great, then she was more harmed by being deprived of those lessons in comparison to some other child who had very little, if any, potential in the area of music).

The point of these examples is to illustrate that Bassen was simply incorrect when he claims that future prospects do not create a present sake, for all of the above examples indicate otherwise. A being's potential can certainly play a pivotal role in deciding how she should be treated or, more precisely for the topic at hand, what moral rights should be allocated to her. While it is true that a potential X cannot be given the rights of an actual X *in certain instances *(when being an actual X is a necessary condition in order to have specific rights), this is not a universal rule, as the above examples illustrate. Potentially possessing a property may be sufficient for entailing an interest in some moral right if possessing that moral right constitutes a benefit for the individual that potentially possesses that property and a denial of that moral right constitutes a harm (e.g., it is not necessary that one actually be sick in order to possess a right to health insurance; potentially possessing the property of being sick is sufficient for entailing a moral right to health insurance, since possessing this right constitutes a benefit, and its denial constitutes a harm, for the potentially sick as well as for the actually sick). From this we can arrive at the following generalization:

##### The moral relevancy of potential principle

A potential X may be granted the same moral rights as an actual X in virtue of its potential if its potential generates an interest in such a moral right; that is, *if possessing the moral right constitutes a benefit for the potential X and a denial of the moral right constitutes a harm*.

It is important to stress the caveat that the potential X must both benefit from the extension of the right of an actual X and also be harmed by its denial. This is because someone may argue that extending the rights of an actual king onto a potential king may indeed benefit him, for I am sure both Charles and William would benefit from the rights and benefits the current queen possesses. Potential physicians, medical students, would certainly benefit from being treated as actual physicians, and so someone may argue that this is sufficient grounds for extending the rights of actual physicians onto them. But in these examples, the potential Xs would not be harmed by a denial of these rights, and so there is no pressing need to accord them these rights why they are still in their potential state. This is not the case for the examples I mention that do warrant extending the rights of actual Xs onto potential Xs. In these cases, a subsequent harm would befall the potential Xs if denied the rights of actual Xs while still in a potential state. The same goes for a right to life. As I will argue below, not only is extending such the right to life onto a potential person a benefit to him, but its deprivation is also a very grave harm, as it would be for an actual person.

Moral rights (and legal rights as well) exist for the *very purpose *of protecting the well-being and welfare of individuals. While I certainly do not have a moral right to anything whatsoever that is in my interests (e.g., I may have an interest in obtaining the money in your wallet, but this does not grant me a moral right to the money in your wallet), moral rights are there to, at the very least, protect our very important welfare interests, the interests that would result in a serious harm if violated. Of course, the respective interests in continued health and continued existence are two of the most important welfare interests there are, and so it can be argued that our moral right to health care and our moral right to life are grounded upon the premise that these integral welfare interests are to be protected as much as possible for all the members of our moral community. If possessing a potential property can make a being a proper subject of harm in virtue of that potential, then this should suffice to extend to her a moral right that would protect her interest in not being harmed and helps to ensure her welfare.

The interest in continued existence is quite possibility the most important welfare interest any individual possesses. As Feinberg writes:

Indeed, there is nothing a normal person... dreads more than his own death, and that dread, in the vast majority of cases, is as rational as it is unavoidable, for unless we continue alive, we have no chance whatever of achieving those goals that are the ground of out ultimate interest [22].

*Contra *Singer, there seems to be no reason to hold that only actual persons have an interest in continued existence. Singer argues that one must possess the capacity to desire continued existence in order to have an interest in it. Not only does this render fetuses incapable of possessing this interest, it renders infants and very young children (e.g., toddlers) incapable as well, since they too lack the conceptual capacity necessary to desire continued existence (and Singer is infamous for his argument that, because of this reason, there is nothing intrinsically morally impermissible about infanticide).

Certainly, however, this flies in the face of commonsense. Most of us hold that infants and young toddlers certainly do have a welfare interest in continued existence, despite their lack of personhood and therefore their inability to desire continued existence. That is, many of us hold that the interest in continued existence is a wholly objective, rather than a subjective, welfare interest. A terminally ill infant, for example, certainly possesses a welfare interest in continued existence, which in turn grounds a *prima facie *moral right to procure a life-saving operation. It would be dubious, to say the least, to argue that it is permissible to let an infant die, when her defect can be easily repaired, on the grounds that she has no interest in the operation or her continued existence because she is utterly incapable of desiring it. As Stone puts it: " [a]n infant need not desire a welfare to have one [23]."

Yet I submit that the reason they have such an interest is strictly in virtue of their potential to become persons. If an infant was afflicted with some horrible defect that rendered her incapable of ever growing past the mental age of a few months old, many would hold that her interest in continued existence would vanquish, or at least would be rendered so weak it would almost be negligible. This is so because death would not harm her as much, if at all, when she has no hopes of ever mentally evolving past a few months old; we would be depriving her of very little by allowing her death, whereas we would be depriving a healthy infant of much more if we killed her, given the enriching life typical to persons. The welfare interest in continued existence is wholly objective rather than subjective, but when it comes to nonpersons, such as infants and young toddlers, their welfare interest in continued existence is based on their potential to become persons and live the rewarding life common to persons. The fact that we usually regard the killing of healthy infants as murder, and the fact that we seem to have no moral qualms or objections against bestowing medical treatment upon infants so that they can continue living their lives and realizing their potential, illustrates that potential does matter. At least when it comes to infants, their potential to become persons certainly influences their *current *welfare interest in continued existence, which, in turn, grounds an interest in medical care and leads to the moral (and legal) judgment of infanticide as a form of murder. (There does seem to be a problem with this claim when we consider whether or not a mentally disabled infant, who will never really grow to have the robust mental capacities of a person, has an interest in continued existence. My claim does seem to, *prima facie*, entail that they lack such an interest, and this may indeed pose a problem given that mentally disabled individuals who are not persons, nevertheless, may experience a life of subjective, although perhaps rudimentary, pleasures. The best response I have for this problem, at the moment, is the following. It is the case that mental disabilities come in degrees, and some individuals with mental disabilities approximate personhood more than others. The strength of the interest in continued existence that a disabled infant possesses may run parallel to how closely she can approximate personhood in the future. As abovementioned, if she has a disease that rendered her unable to ever surpass the mental age of a few months old, her interest in continued existence would seem to be much weaker than the interest in continued existence that a healthy infant possesses. Here, I can appeal to Marquis and his "future-like-ours" view. Perhaps an interest in continued existence is only as strong as how much an infant's future is "like ours", i.e., like a person's. I do want to point out, however, that if we do want to argue that even rudimentary subjective pleasures is sufficient to establish some robust interest in continued existence, we should be willing to grant this interest to all nonhuman animals who experience rudimentary subjective pleasures, lest we concede to speciesism).

Thus, potential can be relevant when it comes to ascribing present interests to some beings in some situations. We can now re-state the pro-potentialist's argument as follows.

**Premise 1**: All innocent persons have a moral right to life.

**Premise 2**: Since all innocent persons have a moral right to life, all potential innocent persons also have a moral right to life.

[*Justification for premise 2*: A moral right to life would constitute a benefit for a potential person as well as for an actual person, and its denial would constitute a harm for a potential person as well as for an actual person].

**Premise 3**: The fetus is a potential innocent person.

**Conclusion**: The fetus has a moral right to life.

This is the justification I believe a pro-potentialist ought to give in defense of premise two. The pro-potentialist can argue that the anti-potentialist's rejection of premise two is a result of his tacit assumption that only actual persons can qualify as bearers of a moral right to life; a claim that the pro-potentialist rejects. As the above examples are meant to illustrate, it seems perfectly justified to treat a potential X as an actual X *if *the potential X has an interest in such treatment; *if *doing so constitutes a benefit for the being in question and a denial of that treatment constitutes a harm. It is my potential to become sick that grounds my current interest, and my moral right, to health insurance even though I am not actually sick; it is a young child's potential to become a rational and moral agent that grounds her current interest in academic and moral education, even though she may not currently be a rational or a moral agent. In all these instances, the potential being possesses some sort of current or actual interest in virtue of her potential, and thus a moral right can be properly be bestowed upon her, in virtue of her potential, in order to protect that interest and, in turn, her well-being and welfare.

So, we have seen that, at times, potential matters and that at other times it does not matter. Does potential matter when it comes to how we ought to treat or regard the human fetus? I think that an argument in the affirmative has best been made by Jim Stone in his articles "Why Potentiality Matters" (1987) and "Why Potentiality Still Matters" (1994). Stone argues that the human embryo or fetus is a being that is intrinsically determined, due to its biological nature, to become a being whose life contains a set of great conscious goods. That is, the embryo's biological nature as a member of the species *Homo sapiens *is of the type that contains its own causal powers that leads to, as Stone puts it, the embryo "mak [ing] itself self-aware [24]." Because the embryo's biological nature is "sufficient to realize self-awareness, social interaction, and the possibility of moral stature," this alone grounds the embryo's claim to "care and protection [25]." Stone's main premise in his argument is that

[a]nything benefits from having the good which it is its nature to make for itself. I submit that we have a prima facie duty to creatures not to deprive them of the conscious goods which it is in their nature to realize [26].

Because the embryo or fetus has a biological nature that, if left to its natural progression, will result in a great good for the embryo or fetus, there is a basis for grounding an interest in continued existence to the embryo or fetus; the embryo or fetus has an interest in continuing to function and realizing its biological nature, a nature that typically brings with it a set of conscious goods that are valuable to possess.

If Stone is right, then the human embryo or fetus possesses a welfare interest in continued existence because its potential as a future person tells us something about what the embryo or fetus is *now*. A fetus' potential is a marker of what it is in its biological nature to achieve, and this, in turn, grounds a welfare interest in continuing life so that it may achieve these conscious goods. Thus, a fetus' potential informs us about what type of future it has *now*, and so the type of future of which it is deprived if not allowed to achieve the conscious goods that it is part of its nature to achieve. As Stone writes:

... if the developmental path determined by the creature's genetic constitution leads to a conscious good for her the creature has an *actual *interest in growing up. It is true for her at t that growing up is a benefit and not growing up is a harm. A creature's present interests are relevant to her rights; therefore potentiality matters [27].

Thus, the fetus' potential can play an important role in the abortion debate. The fetus' potential to become a person indicates what type of future life it will come to possess if allowed to grow up, and this, in turn, grounds a *current *interest in continued existence so that the fetus may realize that future. This interest that may be protected by extending to the fetus at least a *prima facie *moral right to life.

I can foresee two possible objections to my argument, the second of which can serve to segue into the next section of the paper. The first objection is the following. Perhaps I should view my obligation to give my child a good academic and moral education as an obligation to the adult the child will become, rather than to the child at her present state. That is, if I deprive my child of an education *now *there will be a future person, my child *qua *adult, who will be substantially worse off than she would have been had I given her a proper education. Similarly, if I deprive my gifted child of music lessons now, the real victim is not the current child, but rather the adult the child will become who was deprived of the opportunity to become a wonderful musician. Thus, my offspring's potential does not serve to render her *qua *child a victim, but rather her *qua *adult a victim if her potential is not realized. Potential, therefore, does not ground any *current *interests at all. By the same token, the fetus' potential for personhood does not ground any *current *interest for the fetus. The real subject of harm is the future person, who does not actually come into existence if the fetus is aborted.

This leads to the second objection. I can harm the future person that the fetus becomes if I do something now against the fetus, for example, I can administer to a pregnant woman a drug that would result in the fetus' eyes not developing correctly, thereby blinding the future person that develops from the fetus. That is, once the fetus is born there is an individual (the subsequent infant, child, and adult) who is substantially worse off than she otherwise would have been had the development of her eyes not been interfered with while in the fetal stage. But notice, the objection continues, that this is not what happens when we are talking about abortion. If a fetus is aborted, what we are doing is *preventing *the existence of a future person rather than partaking in a current action that will result in a harm for a future person. Thus, when we abort the fetus, we are really harming no currently existing being and we are doing nothing but preventing the existence of a future being. Whereas if we thwart the development of a fetus' eyes, we are, thereby, truly harming someone: the future person that will be blinded as a result, granting that the fetus is allowed to be born and grow up.

The upshot of these two objections is essentially the following: the reason I have to worry about bestowing proper education or medical insurance upon my child in virtue of her potential is because there will be a future being that would be a victim of that deprivation. However, no such future victim exists if the fetus is killed. So, potential matters in the first set of cases I have described because we are dealing with the welfare of a future individual that will suffer as a result of the current child's deprivation. But if I kill a fetus now and stop it from realizing its potential, then there will be no such individual that will be harmed in the future.

Although the two objections are related, I will discuss each one of them distinctly in turn. When it comes to the first objection, which holds that my obligations are really to my offspring *qua *adult rather than my offspring *qua *child, and thus potential really does not ground *current *interests after all, I offer the following two responses. First, once a being exists that can be identified with a future being that experiences conscious goods in her future, then realizing that future is in the *current *being's interest, and not just in the interest of the future being she will become, because that future does not belong to some random other; rather that future is *her *future, and a being has an interest in realizing a future that is full of great conscious goods for her. Indeed she has a welfare interest in such a future, for without being allowed the opportunity to live out her life, any other possible interest she may possess is thwarted. The adult human person and the infant or child from whom she emerged *is the same individual *and thus, it makes little sense to discuss them as if they were two separate entities. As Michael Lockwood puts it: "the infant that [I] once was *had*, I want to say, a strong prospective interest in developing further, seeing that the kind of life [I have enjoyed] constitutes a great benefit, and that the infant and later [me] *are one and the same individual *[28]."

Second, even if I grant that the proper subject of my moral obligation is to my offspring *qua *adult, rather than to my offspring *qua *child, this really makes no practical difference in how I treat my child who is currently at a young stage because, again, *they are the same individual; they share an identity relation*, and therefore I could not fulfill my obligation to my adult offspring without, in a sense, "going through" my young offspring. If I have an obligation to my adult offspring to ensure that she is an educated person, I cannot fulfill this obligation without educating her as a child. If I have a moral obligation to ensure that my offspring grows into adulthood so that she can experience a valuable life, then I cannot fulfill this obligation without ensuring that her welfare interest in continued existence is respected throughout all the stages of her life.

The second objection can be read in two ways. If the fetus is *not yet identical *to any future person, if there is no identity relation between the currently existing fetus and some future person, then it is true that all abortion does is deprive a possible person from coming into existence rather than harming a currently existing individual. Unless one wants to declare contraception or abstinence *prima facie *morally wrong, however, it must be conceded that there is no obligation to bring possible people into existence. Yet, if the fetus shares an identity relation with a future person so that the person's life constitutes the fetus' future, then the objection is not at all effective. It would be tantamount to arguing that it is morally impermissible to harm a future person by inflicting a current damage that will affect her in her future state (e.g., blinding a fetus so that the future person will suffer from blindness), but that harm can be circumvented by ridding the world of the being altogether; a being that *already exists*, an *actual *being, who would grow up to experience all the conscious goods common to the life of persons. If this is correct, however, then I am hard-pressed to find any reason why killing a being would ever constitute a moral harm against it, unless we reduce the moral harm of killing a person to simply thwarting her desire for continued existence, which would render the interest in continued existence a subjective, rather than objective, interest. It certainly seems dubious to argue that we can evade the future victimization of a being by killing the being now, an individual that actually exists and who's future you are taking away, so he will not exist in the future when harm may have taken place. I do not evade the moral wrongness of blinding my baby by killing her so that she will not have to suffer from being blind. If anything, I do nothing but add much insult to injury

But notice that the whole discussion demands that we antecedently answer the question of personal identity. If the fetus is nothing more than the material precursor to an actual human life, so that there is really no identity relation between the currently existing fetus and a future person, then abortion would be tantamount to birth control in the sense that it only prevents the existence of an individual. However, if one thinks that the fetus already *is *the same individual as the future person, then killing the fetus is not preventing the existence of anyone, it is, rather, killing someone that already exists and as such is tantamount to killing an infant or a young child who would have, if not killed, grown into a person. For example, because Sumner holds that the attainment of sentience is such a pivotal threshold for a fetus to cross, he argues that

[e]arly (prethreshold) abortions share the former category with the use of contraceptives, whereas late (postthreshold) abortions share the latter category with infanticide and other forms of homicide... after the [fetus crosses the] threshold [of sentience] there is such a creature, and its normal future is rich and full of life. To lose that life is to sustain an enormous loss [29].

For Sumner, human life begins to exist in all relevant ways, in a way that grants what he calls "moral standing" to the fetus, upon the acquisition of the capacity for sentience and consciousness. It is at this point, then, that potential begins to matter for Sumner, for now there is a being with moral standing that stands to gain from that potential developing and concurrently stands to lose from that potential being frustrated. Before then, however, there is no such being; all that exists before then is a merely possible being, according to Sumner.

I will take it as a given that we are under no moral obligation to respect the interests of only possible people simply because possible people can, at best, have only possible interests. If an individual life has not yet come into existence, if it is only a possible being, then we cannot speak of respecting *actual *interests. Therefore, we cannot address the issue of the moral importance of fetal potential until we first address the issue of personal identity. That is, we must first determine when a being begins to exist who is identical to a future person, thereby making it the case that the current individual possesses an actual welfare interest in not being killed so that she can realize her potential to become that person.

### When does fetal potential become morally relevant?

#### The necessity of addressing the issue of personal identity

Potential certainly seems to matter, and it certainly seems to matter when it comes to the human fetus if one thinks that the fetus is a being that is identical to a future being who will experience the conscious goods typical to persons and thus has an interest in realizing those goods. Does this mean that my argument leads to a rather conservative position against abortion? It may or it may not; this question cannot really be answered until we first address the question of personal identity. In other words, the answer to this very complex practical moral problem has its roots in the intricate metaphysical issue of when a being's identity begins to exist. Jim Stone captures this importance quite well when he asserts that: " [a] human creature's moral right to life begins when he does [30]."

When does potential matter? I would like to adopt Michael Lockwood's claim that " [a] potential for X generates an interest only where there is some individual for whom the development of the potential for X constitutes a *benefit *[31]." That is, in order to meaningfully discuss the benefits of realizing a being's potential, the being in question must be more than a purely possible being, since possible beings can, at most, have only potential interests and not actual ones. That is, the being must *actually exist *in order to possess a current and *actual *interest in realizing that potential (this does not mean that the being must exist *right now *in order to have some sort of interest. As Lockwood argues, the actual existence of this being can be understood in a tenseless fashion, a being that exists at any time, either past, present or future. If we know that a being is going to certainly exist in the future, then we can certainly thwart its interests now by doing something that will be detrimental for him in the future) [32].

One reason why it is so important to stress that potential only begins to matter when there is a being in existence of whom the realizing of that potential would constitute a benefit is because doing so allows a response to the sperm/ova problem. Although both a sperm and an ovum each possesses some potential, if united, to produce a being who will grow up to become a person, neither of them possess the potential on their own. At conception, the sperm and the ovum lose their own numerical identity and together give rise to a wholly new being: a being that possesses the genetic constitution of the sperm and the genetic constitution of the ovum, but is not identical to either. Gametes produce a new being, but neither of them, themselves, are this new being and so the being who would come to benefit from the realization of potential does not exist before the gametes unite. Don Marquis, for example, argues that before conception takes place, there does not yet exist a subject to whom we can attribute a valuable future. Gametes themselves do not possess a valuable future because they cease to exist upon fusion and a new ontologically distinct being, the zygote, begins to exist.

Prior to conception there is no individual that is the same individual as the later human being that has, or would have had, a valuable life. Individual identity does not survive fusion or fission, whether contraception, amoeba reproduction, or brain bisection are the examples [33].

Stone also holds to a similar view.

The sperm and the egg cannot both be identical to the adult human being, nor can that creature be identical to the adult human being, nor can that creature be identical to just one of them... [b]oth the sperm and the egg can produce something which has the potential of becoming an adult human being, but neither the sperm nor the egg has that potential itself [34].

The kind of potential harbored by gametes to become persons is of a different type than the potential harbored by infants, children, and perhaps even fetuses. In order to more clearly distinguish between these two different types of potential, Stone denotes them differently. There is what he calls "strong potential," which is identity preserving potential. If A has the strong potential to become B, then "A will produce B if A develops normally and the B so produced will be such that it was once A [35]." Then there is what he calls "weak potential," which is non-identity preserving potential. To say that A has the weak potential to become B is to say that A can help in the production of B, but A itself will not become B. That is, A is "an element in a causal condition that produces B and, further, the matter of A will be (or will at least help produce) the matter of the B [36]." A child, for example, has the strong potential to become an adult person because the adult and the child are *the same individual*; they have an identity relation. Gametes, on the other hand, have only the weak potential to become an adult person because their respective matter will help produce the future adult, but neither of them is already the individual that will become that adult. There is no identity relation between an adult person and the gametes that produced her, if for no other reason than because they are numerically different: gametes are two distinct beings in their own right while the adult human is a single distinct being.

It is strong potential that matters when it comes to this issue. Since a child has the strong potential to become an adult, that adult's life already forms part of the child's *current *interests in growing up, since it *his *future that we are talking about. Gametes, however, do not possess an identity relation with that future being and so the best we can do is discuss the *possible *interests of the *possible *individual that *may *come into fruitarian *if *the gametes unite. There is no actual interest yet because there is no actual subject yet, and thus no subject of whom the realization of potential would constitute a benefit. Once an individual begins to exist that is the same individual as a later being, then we can meaningfully say that there is an individual currently in existence that would benefit from being allowed to develop that potential. When all we have are gametes, there is no subject yet in existence that would benefit from being allowed to grow up, and, therefore, there is no such victim yet that is being deprived of that opportunity. As Stone writes:

If we kill the fetus we deprive her of a welfare she would have otherwise have realized for herself. The sperm and the egg, on the other hand, can never have these properties even though they can produce something which can. If we kill them there is no good of which they are deprived [37].

So when does there begin to exist a subject that is identical to a future being, so that realizing that future being's life grounds a current interest in that subject growing up? The answer to this question will determine when strong potential begins to exist and thus when there is a current actual interest in continued existence. I will not endorse any one view of personal identity here, since defending one view is a long and complex thesis in its own right, and would take us beyond the scope of this paper. Instead, what I will do is offer a brief survey of three accounts of personal identity in order to illustrate how pivotal the question is before we can find the proper place of potential when it comes to attributing to a fetus a welfare interest in continued existence.

##### Animalism

Animalism is the philosophical view of personal identity, famously defended by Eric Olson [38], that states that people continue to exist over time so long as their numerically distinct organism continues to exist. According to this theory, you are essentially a human animal, you persist as long as your organism does, and you come into existence whenever your numerically distinct organism comes into existence. According to some philosophers, however, this does not occur until approximately fourteen days post-fertilization, when the cells in the zygote no longer divide into identical daughter cells, and when multiple ontologically distinct zygotes can no longer emerge (which is how multiple births come to be). For example, philosopher and theologian Normal Ford argues that "it would be hard to admit the presence of an individual human being in the zygote if, in principle, it could lose its ontological identity whenever twinning occurs in the course of development [39]." Furthermore, Karen Dawson writes:

... the individual created at fertilization may not remain the same throughout life. The simplest demonstration of this is identical twinning which is possible for about 12 days after fertilization. In this process the original zygote ceases to exist. Conversely, during this time it is also possible for two zygotes derived from the independent fertilization of two eggs to fuse forming a chimera – the one individual resulting from two fertilization events [40].

According to Animalsim, then, we begin to exist not when our unique genetic code comes into existence, not at conception, but rather when our numerically distinct organism begins to exist, at approximately fourteen days post-fertilization, when division is no longer possible and thus where is there irreversible biological identity. After this time, however, we have a distinct human animal that is identical to a future human animal that will live the type of life common to persons. Thus, according to Animalism, human identity begins rather early in pregnancy. Coupled with the ethical considerations of potential outlined above, such a view of personal identity *may *lead to a rather conservative view on abortion because there is an identity relation between what is now an embryo (the fertilized egg after two weeks), and the subsequent person the embryo will become in virtue of the fact that they are the same human animal. According to someone who holds to Animalism, the embryo has the strong potential to become that adult, and thus the embryo, and the subsequent fetus, can be properly ascribed an interest in continued existence (I want to emphasize the term "may" because it is still possible to maintain that the fetus has a welfare interest in continued existence, in virtue of being the same human animal as a future person that will live a valuable life, but still hold that abortion is morally permissible. The reason for this is that it has yet to be established whether the fetus' interest in continued existence trumps a woman's right to bodily autonomy. This is an entirely separate question, however, and, other than briefly mentioning it again below, it is not an issue that I will deal with in this paper. Moreover, it does not necessarily follow that the beginning of an identity relation with a future person entails the beginning of moral status. For example, David DeGrazia separates the two in his recent book *Human Identity and Bioethics *[41]. Although he does endorse a version of Animalism in his book, he does not argue that moral status begins whenever numerical identity begins).

##### Psychological Criterion Account of personal identity

Animalism, however, is rejected by many. Some philosophers argue that personal identity is intimately correlated in some sense with possessing some sort of mental life, although many differ as to how robust that mental life must be in order to establish an identity relation. The view that we are essentially *persons*, and thus that we comes into existence as persons, persists as persons, and die when we lose our personhood, is called the Psychological Criterion Account of Personal Identity. For example, Mary Ann Warren argues that:

personal pronouns like "we" refer to people; we are essentially people if we are essentially anything at all. Therefore, if fetuses and gametes are not people, then we were never fetuses and gametes, though one might say that we emerged from them. The fetus which later became you was not *you *because you did not exist at that time... [s]o if it had been aborted nothing whatever would have been done to you, since you would have never existed [42].

Notice that Warren also makes the assumption that a being must exist, in some way, before she can be the proper subject of harm or benefit. If we were never identical to a fetus, if, in fact, we came into existence gradually as our personhood gradually arose, then abortion would not have harmed *us *because we had not yet existed in the fetal stage. In this sense, Warren may perhaps agree with Lockwood that the realization of potential is only morally relevant when there exists a being of whom the realization of that potential constitutes a benefit. But, according to Warren, no such individual yet exists at the fetal stage because the fetus is not identical to any future person who experiences life. Thus, there exists no one, at the fetal stage, who would benefit from the realization of potential. Peter Singer, who also maintains that it is necessary to be an actual person in order to have an interest in continued existence and a moral right to it, also seems to espouse a similar view of personal identity. He writes:

I am not the infant from whom I developed. The infant could not look forward to developing into the kind of being that I am, or even into any intermediate being, between the being I now am and the infant. I cannot even recall being the infant; there are no mental links between us [43].

Singer seems to hold that in order for there to be an identity relation between the infant from whom I developed and myself currently, the infant must have been able to conceive of herself as a future person and must have had the capacity to "look forward" to becoming that future person. In other words, self-consciousness, in addition to memory retention, seems to be a necessary condition, according to Singer, for possessing any type of significant mental links that could establish an identity relation. Like Warren, therefore, Singer seems to be arguing that an identity relation between past and future stages of a self can only come into being once personhood arises, for only persons possess self-consciousness in the robust fashion that Singer believes is requisite for an identity relation.

The plausibility of the Psychological Criterion Account is not the subject of this paper, and so I will neither defend nor criticize it. What is important to see, however, is the upshot of accepting this theory when it comes to the question of the importance of fetal potential. If we are essentially persons, and thus we do not really begin to exist until there is a person on the scene, fetuses, then, possess only the weak potential to produce persons, i.e., their matter will contribute to the making of a future person, but they will not *become *persons themselves. On this view, then, fetuses lack identity-preserving potential. Although Stone disagrees with the Psychological Criterion Account himself, he succinctly sums up the consequences of holding to such a view.

Warren's view is that the being which realizes self-awareness is a person; a person comes into being when it realizes self-awareness; hence a person was never a fetus or an infant. It follows that the fetus, like the sperm, produces a numerically different entity which is the thing that thinks and feels, so the fetus has no welfare of its own [44].

Therefore, according to this view, potential never counts throughout the fetal stage and no fetus ever has an interest in continued existence. Of course, this also leads to the conclusion that any potential that a neonate possesses is also non-identity preserving potential, since neonates are not persons yet. Thus, killing a neonate does not deprive her of her future simply because she has no such future yet, since she is not yet identical to any future person. This would make the killing of a neonate intrinsically morally negligible, since all we are doing is preventing a future person from coming into existence, rather than killing an actually existing one, which is a position that Warren herself admits to [45].

##### Embodied Mind Account of personal identity

This account of personal identity also holds that possessing some sort of mental life is necessary for identity to exist and persist over time. However, the degree of mental complexity that is requisite is no where near the robustness that the Psychological Criterion Account requires. According to the Embodied Mind Account, a human being begins to exists in all the ways that matter, in a way that allows her to be identified with a future being, when she gains the capacity for conscious awareness sometime during fetushood (at approximately mid-gestation). Jeff McMahan is one such defender of this view.

I suggest that the corresponding criterion for personal identity is the continued existence and functioning, in nonbranching form, of enough of the same brain to be capable of generating mental activity. This criterion stresses the survival of one's basic psychological capacities, in particular the capacity for consciousness. It does not require continuity of any particular *contents *of one's mental life [46].

Michael Lockwood also seems to adhere to this theory, although he does not refer to it by this title. Nevertheless, he argues that

[w]hat our identity actually consist in, I suggest, is, once again, whatever as a matter of scientific or metaphysical fact, normally underlies these more superficial continuities and provides the deep explanation for them; and from a purely secular standpoint I should have thought that the overwhelming most favored candidate was a continuity of organisation within those parts of our brain that directly sustain those activities we think of as mental... considerations of identity firmly favor the view that before the brain has matured to the point of being able to sustain psychological functions, a human life has yet to begin [47].

Advocates of this view, then, maintain that no human identity really begins to exist until the fetus becomes capable of consciousness awareness. Before this point, the human fetus possesses a biological life, but not a biographical one. A biographical life occurs once the fetus becomes capable of having some sort of inner mental life, when it becomes a locus of consciousness. Persistence of identity does not necessitate a robust form of self-consciousness or rationality, as the Psychological Criterion Account seems to hold, but it does necessitate at least *some *form of mental life, even if it is a comparatively rudimentary one.

As a result of this view, the fetus' potential begins to matter in terms of attributing to it an interest in continued existence when it becomes the type of being whose brain can sustain the capacity for conscious awareness, for it is here when an identity relation with a future person begins to exist, and thus it is here when we can attribute the life of this future person as rightfully the fetus' future. McMahan holds, therefore, that "just prior to twenty weeks [the approximate gestational age when a fetus acquires the capacity for consciousness awareness], there is no one there to be affected by an abortion. After twenty-eight weeks, the developed fetus is definitely present and would be affected for the worse by an abortion [48]." Along the same vein, Lockwood writes:

If this is right, then the potential of a human embryo for developing into a [p]erson does not confer on it any right to protection. For it has no brain at all. Consequently, that which would stand to benefit by the development of this potential *does not yet exist *[49].

What is interesting to notice is that none of the abovementioned philosophers really denies that potential never matters, not even Warren who perhaps has the most liberal abortion view and who also, not coincidentally I think, pushes identity much further into biological life than is commonsensical (most of us do think that there is an identity relation between us as adults and our infant self). I say this because I suspect that Warren herself would agree with me that in the examples stated in the previous section, potential does seem to matter and does indeed ground interests: it is because a child is a potential moral agent that her current moral education matters, it is because she is a potential rational agent that her current academic education matters. Potential does matter, but the question is *when *does it begin to matter, and it is *here *where disagreements arise. I fully endorse Lockwood's view that "it is potential plus identity that morally counts for something [50]." The disagreement arises when there are conflicting views concerning when personal identity begins to exist. If one thinks that personal identity consists the persistence of the numerically single human organism, i.e., Animalism, then potential counts rather early in gestation, while still in the embryonic stage, whenever irreversible numerical identity is established. If one holds to the Psychological Criterion Account, potential never matters throughout gestation, since personal identity consists in being the same person over time, and a person comes into existence only after post-infancy. The Embodied Mind Account is the middle ground, which states that personal identity begins in mid-gestation, and therefore, for anyone who holds this view, potential may begin to matter only then.

## Conclusion

Nothing I have said in this paper necessarily grounds a position that abortion is always morally wrong or unjust (which is why I keep referring as the fetal moral right to life as a *prima facie *right). Even though potential can ground an interest in continued existence for an early embryo or mid-gestation fetus, depending on what theory of personal identity one adheres, we still have to contend with Thomsonian-like objections which state that a fetus' moral right to life does not entail a woman's obligation to sacrifice her body in order to gestate it for nine months [51]. Nevertheless, I believe I have demonstrated why potential does matter, and I hope to have also illustrated that perhaps the major disagreement about this issue has more of its roots in the metaphysical question of personal identity that has previously been acknowledged.

Even the most basic and pervasive ethical debate, like the abortion debate and the debate concerning the moral relevancy of fetal potential, is intimately connected with underlying metaphysical assumptions, even though these assumptions are rarely discussed amongst applied ethicists. This illustrates that the tendency to divorce "traditional" or "abstract" philosophy from "practical" philosophy is unfortunate and misguided. In this case, we cannot address the issue of when fetal potential becomes morally relevant until we first address the matter of personal identity, and this is a subject that is very thorny indeed. Although I have only given a brief synopsis of three major views, there are many others, each as highly contested by some philosophers as the next. However, by pointing out the underlying metaphysical assumptions in this area of applied ethics, I hope to have at least nudged the debate further in a new and, with any luck, effective direction.
